# Contributions to the Pathological Museum of the Medical Institute of Louisville

**Published:** 1841-07

**Authors:** 


### THE WESTERN JOURNAL

Vol. IV.—No. I.

LOUISVILLE, JULY 1, 1841.

### CONTRIBUTIONS TO THE PATHOLOGICAL MUSEUM OF THE MEDICAL INSTITUTE OF LOUISVILLE.

We should have apologized for not having at an earlier period acknowledged the receipt of the following specimens, many of which are valuable. It will, we hope, be gratifying to the friends of science who sent them, to know that they are used in the illustration of the lectures on Pathological Anatomy, Surgery, and Obstetrics, in the Institute, and thus made to contribute to the education of the students of the West. We return our thanks to the doners, and respectfully solicit from them, and their brethren, additional contributions. They will be permanently preserved and labelled with the names of those who send them; and an account of the cases kept in a register provided for that purpose.

### KENTUCKY.

From Dr. John C. Brent, late of *Louisville.*

A large, firm adipose tumour, successfully extirpated, by himself, from the neck of a lady.

A polypus of the gelatinoid variety, taken by himself from the nose of a gentleman.

From Dr. Samuel B. Richardson of the same city.

A specimen of carcinomatous liver, the disease extending to the gall bladder which contained several *black* biliary calculi.

A gelatinous polypus, extracted by himself from the nose of a young lady.

From Dr. William T. Taliaferro, of *Maysville.*

A fibrous uterine polypus, removed by himself.

From Dr. John M. Duke, of the same city.

A heart showing the ravages of pericarditis.

### OHIO.

From the late Dr. Dudley W. Rhodes of *Zanesville.*

A very large encephaloid tumour, from the upper part of the cavity of the abdomen. We may hereafter give a history of the case.

A testis and epidydimus, enlarged and indurated by chronic inflammation. Successfully extirpated by himself.

An osseous specimen from an aged female, in which the entire neck of the os femoris had been absorbed.

From Dr. J. J. Brice, of *Newark.*

A specimen of strongylus, six inches long, discharged from the bladder of a female patient.

From Dr. John W. Russell, of *Mount Vernon.*

Four specimens of urinary calculi, extracted by himself.

From Dr. John Dawson, Jr., of *Jamestown.*

A specimen of intussuscepted ileon, more than two feet long, discharged per anum, his patient recovering.

From Dr. Davius Maxon, of *Gallipolis.*

An uncommonly voluminous adipose tumour extirpated by himself, from the back part of the thorax of a woman.

A scirrhous breast, likewise successfully extirpated by himself.

A pin discharged through the parieties of the abdomen, nineteen years after it had been swallowed.

### INDIANA.

From Doctor Rogers, of *Madison.*

A large fibrous tumour successfully removed by himself from the arm of a child eighteen months old.

### ARKANSAS.

From Dr. P. M. Enders, of *Van Buren.*

A hypertrophied heart with valvular vegetations. D.

